# Association of microalbuminuria with metabolic syndrome: a cross-sectional study in Bangladesh

**DOI:** 10.1186/s12902-020-00634-0

**Published:** 2020-10-07

**Authors:** Muntakim Mahmud Saadi, Manindra Nath Roy, Rubena Haque, Farida Akhter Tania, Shakil Mahmood, Nurshad Ali

**Affiliations:** 1 Department of Biochemistry, Netrokona Medical College, Netrokona, Bangladesh; 2 Department of Biochemistry, Sir Salimullah Medical College, Dhaka, Bangladesh; 3 Department of Biochemistry, Ad-Din Women’s Medical College, Dhaka, Bangladesh; 4 Department of Biochemistry, Gonoshasthaya Samaj Vittik Medical College, Savar, Dhaka, 1344 Bangladesh; 5 Department of Biochemistry and Molecular Biology, Shahjalal University of Science and Technology, Sylhet, 3114 Bangladesh

**Keywords:** Microalbuminuria, Metabolic syndrome, Prevalence, Bangladeshi adults

## Abstract

**Background and aims:**

The objectives of this study were to estimate the prevalence of microalbuminuria and examine the association of microalbuminuria with metabolic syndrome (MetS) and its component in a Bangladeshi adult cohort.

**Methods:**

This cross-sectional study included 175 subjects (84 males and 91 females; aged 19–59 years), recruited from the outdoor Department of Medicine and Endocrinology of a medical college hospital in Dhaka, Bangladesh. Lipid profile and fasting blood glucose (FBG) were measured in serum and albumin and creatinine were determined in urine samples. Microalbuminuria was defined as the urinary albumin-to-creatinine ratio (ACR) of 30 to 300 mg/g. The MetS was defined according to the criteria of the National Cholesterol Education Program (NECP). The association of microalbuminuria with MetS and its components was evaluated by multivariate logistic regression analysis.

**Results:**

Among the study subjects, 66.3% were hypertensive and 70.3% were diabetic individuals. Overall, the prevalence of microalbuminuria was 29.7% with 31% in males and 28.6% in females. Microalbuminuria was 2.6 fold higher in hypertensive and diabetic adults than in the non-hypertensive or non-diabetic adults. The prevalence of microalbuminuria was much more frequent in persons with the MetS (36.0%) than the persons without the MetS (5.4%). The levels of FBG, systolic blood pressure (SBP), diastolic blood pressure (DBP) and triglycerides were significantly higher (*p* < 0.01 for all cases) in subjects with microalbuminuria. In regression analysis, after adjusting for sex, age, and body mass index, microalbuminuria was strongly correlated with MetS followed by elevated BP and FBG (*p* < 0.01 for all cases).

**Conclusions:**

Microalbuminuria was strongly associated with MetS in Bangladeshi adults. Elevated BP and FBG were the most predominant components of MetS among the study subjects. Comprehensive management of MetS at its early stage can be effective to prevent and reduce the progression of kidney injury and cardiovascular complications.

## Background

Microalbuminuria is considered as an early indicator of chronic renal disorder, vascular dysfunction and cardiovascular mortality [1–3]. Microalbuminuria is more frequent in subjects with type 2 diabetes [3] and has been included in the unifying definition of metabolic syndrome (MetS) suggested by the WHO [4]. In previous studies, microalbuminuria was associated with hypertension and abdominal obesity [5, 6]. However, the inclusion of microalbuminuria as an essential component of the MetS remains controversial.

MetS is a group of metabolic abnormalities characterized by elevated blood pressure, hyperglycemia, abdominal obesity, high triglycerides, and reduced high-density lipoprotein cholesterol that collectively increases the risk of diabetes, cardiovascular diseases, and overall mortality [7–9]. The American Heart Association criteria [10] and the Adult Treatment Panel III criteria (ATP III) [11] did not include microalbuminuria as part of MetS. Nonetheless, several studies showed an association of microalbuminuria with MetS and its components in adults [12–16]. For example, in the third National Health Nutritional Examination Survey in the US, microalbuminuria showed an association with the components of MetS; blood pressure and fasting blood glucose [16]. In another study, a positive association has been reported for microalbuminuria with elevated blood pressure, high TG and reduced HDL-C [17].

In the recent years, the prevalence of MetS is rapidly increasing both in developing and developed nations. In Bangladesh, the prevalence of MetS has been increased over the last few decades. A review by Chowdhury et al. [18] reported a high prevalence of MetS (30%) in the Bangladeshi population. A very recent study showed the prevalence of MetS 22% in general adults with 21.9% in males and 22.1% in females [19]. A few studies conducted in Eastern Asia showed a relationship between microalbuminuria and MetS but generated inconsistent results with the components of MetS [12–15]. Therefore, it is important to examine whether microalbuminuria is associated with MetS in general adults. Given the rising prevalence of MetS in Bangladeshi adults, this cross-sectional study was conducted to examine the relationship of microalbuminuria with MetS and its components in a Bagladeshi adult cohort.

## Methods

### Study population

This cross-sectional study was conducted at the Department of Biochemistry of Sir Salimullah Medical College, Dhaka, Bangladesh. The study participants (*n* = 175) were enrolled from the outdoor Department of Medicine and Endocrinology of Mitford Hospital, Dhaka, Bangladesh from March 2017 to January 2018. The participants underwent the Department for their physical checkup and treatment. The preliminary inclusion criteria were both genders, aged ≥18 years, free from chronic diseases such as stroke, arthritis and cancer. However, lactating mothers, pregnant women, and subjects with a history of alcohol consumption, hepatotoxic drug intake, and self-reported indications of acute or chronic hepatitis B infection were excluded from the study. This study was approved by the institutional Ethics Committee of Sir Salimullah Medical College and Hospital, Dhaka, Bangladesh. All study participants were informed about the study aims and we obtained written informed consent from them before inclusion in the study. We followed the institutional guidelines and regulations to perform all steps of the methodology section.

### General data collection

Individual anthropometric data eg. age, gender, height, weight and information on lifestyle factors were included in a short questionnaire form maintaining the standard measure described elsewhere [20–23]. Briefly, body weight was measured to the nearest 0.10 kg by a digital weighing machine (Beurer 700, Germany) and body height was measured to the nearest 0.10 cm by height measuring tape wearing light clothes and no shoes. The body mass index (BMI) was calculated as body weight in killograms divided by height in meters squared [19]. Waist circumference (WC) was measured using a general tape that was placed midway between the lowest border of the ribs and iliac crest. Systolic blood pressure (SBP) and diastolic blood pressure (DBP) were measured by an automated sphygmomanometer (Omron M10, Omron Corporation, Tokyo, Japan) and the average value was used in the calculation [24–26]. The accuracy of the obtained data was confirmed by repeated-measures analysis.

### Blood and urine sample collection and laboratory analysis

The venous blood samples were obtained after 10-12 h overnight fasting from the participants [19]. Then the samples were centrifuged at 3000 rpm for 15 min and isolated serum was stored at -20 °C until analysis. Fasting blood glucose (FBG), triglycerides (TG), total cholesterol (TC), low-density lipoprotein cholesterol (LDL-C) and high-density lipoprotein cholesterol (HDL-C), were measured by standard colorimetric methods. Fasting first-morning void urine samples were collected for the determination of urinary creatinine and albumin concentrations. Urinary creatinine was measured using a kinetic assay applying Jaffe creatinine method and urinary albumin was measured using an immunoturbidimetric method. Urine albumin-creatinine ratios (ACR) were calculated for all subjects. These measurements accuracy were maintained through the standard calibration regularly.

### Diagnostic criteria

MetS was diagnosed based on the criteria of the National Cholesterol Education Program – Adult Treatment Panel III (NCEP-ATP III) [27]. The variables of MetS were defined as follows: i) Elevated blood pressure (SBP ≥130 mmHg and/or DBP ≥85 mmHg or antihypertensive medication intake); ii) raised waist circumference (> 102 cm for men and > 88 cm for women); iii) hypertriglyceridemia (TG ≥150 mg/dL); iv) hyperglycemia (FBG ≥100 mg/dL) and v) low HDL-C (< 40 mg/dL for men and < 50 mg/dl for women) [19] . MetS was identified among the participants who had at least three of the above components. SBP ≥ 140 mmHg and/or DBP ≥ 90 mmHg were considered to define hypertension [28]. The presence of diabetes was confirmed by the American Diabetes Association criteria as the fasting blood plasma glucose level of ≥126 mg/dL or random plasma glucose ≥200 mg/dL [29], or self-reported recent use of hypoglycemic medication. Microalbuminuria was defined as a urinary albumin-to-creatinine ratio (ACR) of 30 to 300 mg/g [16].

### Statistical analysis

Statistical data analyses were done using IBM SPSS version 23. Data are presented as mean ± SD. Categorical variables were analyzed using the Chi-square test. Comparison of the baseline data in the male-female group was done by the independent sample t-test. The relationship between microalbuminuria and levels of baseline variables was determined by Pearson’s correlation coefficient test. Multivariate logistic regression analysis was performed to evaluate the association of microalbuminuria with MetS and its components, after controlling covariates, including sex, age, and BMI. In regression analysis, MetS components were mutually adjusted. *P*-values of < 0.05 were considered as statistically significant.

## Results

The baseline characteristics of the study participants are presented in Table 1. In total, 175 participants (84 males and 91 females) were enrolled in the present study. Males, compared to females, were slightly older. No significant difference was observed for age, BMI, blood pressure, FBG, urinary creatinine and albumin and lipids except HDL-C (*p* < 0.05) between males and females. The prevalence of hypertension and diabetes was 66.3 and 70.3%, respectively among participants. Overall, microalbuminuria was present in 29.7% of subjects with 31% in males and 28.6% in females. The prevalence of microalbuminuria was 41.4% in hypertensive and 35.8% diabetic individuals, and 13.6% in non-diabetic and non-hypertensive individuals. The overall prevalence of MetS was slightly higher in females (82.4%) than in the male (79.8%) participants.

**Table 1:** Anthropometric and metabolic characteristics of the study subjects

Characteristics	Total	Male	Female	*P*-value
N	175	84	91	–
Age (years)	42.6 ± 8.1	43.3 ± 8.2	41.9 ± 8.0	0.293
BMI (kg/m^2^)	27.3 ± 3.4	26.8 ± 3.3	27.8 ± 3.5	0.055
WC (cm)	93.9 ± 7.1	94.3 ± 6.9	93.7 ± 7.4	0.592
SBP (mm Hg)	136.7 ± 22.1	138.3 ± 20.5	135.2 ± 23.5	0.354
DBP (mm Hg)	88.4 ± 10.0	89.4 ± 8.7	87.5 ± 11.1	0.203
FBG (mg/dL)	169.0 ± 59.0	163.7 ± 62.1	173.9 ± 55.8	0.255
TC (mg/dl)	216.1 ± 43.8	210.7 ± 40.5	221.1 ± 46.3	0.117
TG (mg/dL)	198.3 ± 79.7	195.6 ± 66.2	200.7 ± 90.7	0.677
HDL-C (mg/dL)	37.3 ± 5.8	36.3 ± 4.8	38.2 ± 6.4	0.034
LDL-C (mg/dL)	140.1 ± 41.4	136.5 ± 40.0	143.4 ± 42.5	0.275
UA (mg/L)	45.2 ± 54.6	45.6 ± 54.0	44.9 ± 55.5	0.927
UCR (mg/dL)	181.6 ± 92.0	183.9 ± 91.1	179.4 ± 93.2	0.745
ACR (mg/g Cr)	26.6 ± 36.9	24.7 ± 34.7	28.3 ± 38.9	0.526
Hypertension, n (%)	116 (66.3)	59 (70.2)	57 (62.6)	< 0.05
Diabetes, n (%)	123 (70.3)	53 (63.1)	70 (76.9)	< 0.05
Microalbuminuria, n (%)	52 (29.7)	26 (31.0)	26 (28.6)	< 0.05
MetS, n (%)	142 (81.1)	67 (79.8)	75 (82.4)	< 0.05

*BMI* body mass index, *WC* waist circumference, *SBP* systolic blood pressure, *DBP* diastolic blood pressure, *FBG* fasting blood glucose, *TC* total cholesterol, *TG* triglyceride, *HDL-C* high-density lipoprotein cholesterol, *LDL-C* low-density lipoprotein cholesterol, *UA* urinary albumin, *UCR* urinary creatinine, *ACR* urinary albumin to creatinine ratio, *MetS* metabolic syndrome. Data are presented as mean ± SD. *P*-values are obtained from Independent sample t-test in comparison between male-female groups. Chi-Square test was applied to determine *p*-values for categorical variables

**Fig. 1:**
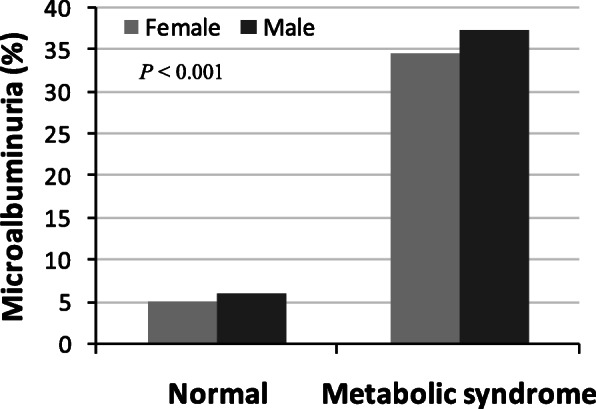
Prevalence of microalbuminuria by metabolic syndrome. *P* < 0.001 when compared the prevalence of microalbuminuria between normal and metabolic syndrome groups

**Fig. 2:**
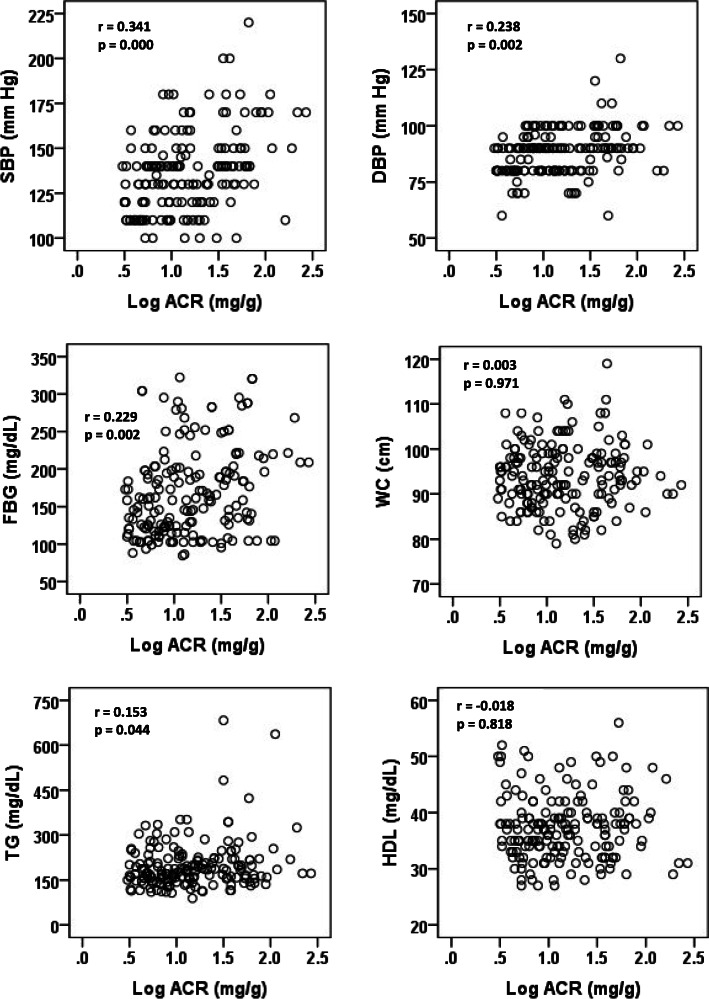
Association of individual component of metabolic syndrome with ACR (log transformed concentrations)

Table 2 shows the anthropometric characteristics and prevalence of each component of the MetS by microalbuminuria status. The levels of FBG, blood pressure and TG were significantly higher (*p* < 0.01 for all cases) in participants with microalbuminuria. Microalbuminuria was much more prevalent in persons with the MetS (36.0%) than persons without the MetS (5.4%) (Fig. 1). The prevalence of microalbuminuria was higher in males with the MetS. The relationship between the individual component of MetS and ACR are presented in Fig. 2. A positive and significant association was observed for ACR with SBP (*p* < 0.001), DBP (*p* < 0.01), FBG (*p* < 0.01) and serum TG (*p* < 0.05) levels. After adjustment of age, sex and BMI in logistic regression analysis, microalbuminuria was significantly associated with elevated BP and high FBG (*p* < 0.01 for both cases) (Table 3). With a similar adjustment of covariates in the regression model, microalbuminuria was strongly associated with the MetS (OR 4.38, *p* < 0.01).

**Table 2:** Anthropometric and metabolic characteristics according to albuminuria status

Characteristics	Normoalbuminuria (< 30 mg/g)	Microalbuminuria 30–300 mg/g	*P*-value
N	123	52	–
Sex, m/f (n)	58/65	26/26	–
Age (years)	41.8 ± 8.0	44.5 ± 8.2	0.045
BMI (kg/m^2^)	27.1 ± 3.4	27.9 ± 3.4	0.171
WC (cm)	93.5 ± 7.1	95.1 ± 7.2	0.166
SBP (mm Hg)	131.1 ± 19.0	149.8 ± 23.4	0.000
DBP (mm Hg)	86.2 ± 8.7	93.7 ± 10.9	0.000
FBG (mg/dL)	160.4 ± 56.1	189.2 ± 61.4	0.003
TC (mg/dl)	215.6 ± 44.3	217.3 ± 42.9	0.812
TG (mg/dL)	186.9 ± 55.7	225.0 ± 114.9	0.004
HDL (mg/dL)	37.1 ± 5.6	37.6 ± 6.2	0.659
LDL (mg/dL)	141.0 ± 44.1	137.7 ± 33.7	0.639
ACR (mg/g Cr)	10.1 ± 6.1	65.5 ± 48.5	0.000
Hypertension, n (%)	68 (55.3)	48 (92.3)	> 0.01
Diabetes, n (%)	79 (64.2)	44 (84.6)	> 0.05

*BMI* body mass index, *WC* waist circumference, *SBP* systolic blood pressure, *DBP* diastolic blood pressure, *FBG* fasting blood glucose, *TC* total cholesterol, *TG* triglyceride, *HDL-C* high-density lipoprotein cholesterol, *LDL-C* low-density lipoprotein cholesterol, *ACR* urinary albumin to creatinine ratio. Data are presented as mean ± SD. *P*-values are obtained from Independent sample t-test in comparison between the groups. Chi-Square test was applied to determine *p*-values for categorical variables

**Table 3:** Association of microalbuminuria with MetS and its components

MetS component	Adjusted OR (95% Cl)	*P*-value
Elevated FBG	2.70 (1.58–3.87)	0.001
Large WC	1.78 (0.66–3.84)	0.420
Elevate BP	3.25 (2.30–4.22)	0.001
Elevated TG	1.96 (0.88–3.12)	0.068
Reduced HDL-C	1.76 (0.60–3.56)	0.328
MetS	4.38 (1.88–6.99)	0.001

*FBG* fasting blood glucose, *WC* waist circumference, *BP* blood pressure, *TG* triglyceride, *HDL-C* high-density lipoprotein cholesterol, *MetS* metabolic syndrome. Multivariate logistic regression analysis was performed to evaluate the association of microalbuminuria with MetS and its components. The model has been adjusted for sex, age and BMI, and additionally for other components of the metabolic syndrome. *OR* odd ratio, *CI* confidence interval

## Discussion

In the present study, we found a strong positive association between microalbuminuria and MetS among the study subjects. Microalbuminuria was common among the participants (29.7%) and much more prevalent in subjects with the MetS (36.0%) than subjects not having the syndrome (5.4%). Of the components of MetS, high FBG and elevated BP were strongly associated with microalbuminuria. The magnitude of the observed association remained even after adjusting for sex, age, BMI and other components of the MetS. Our results indicate that microalbuminuria may be a component of MetS and these findings supporting the results of some previous works [8, 30–34]. To the best of our knowledge, this is the first study that evaluated the relationship between microalbuminuria and MetS among Bangladeshi adults.

The prevalence of microalbuminuria in our study was higher than the studies conducted previously in the Chinese, Japanese and US general population [13, 16, 34]. Where this prevalence was 4.3% in Chinese [34], 13.7% in Japanese [13] and 6.4% in the US population [16]. The differences in the prevalence of microalbuminuria in these populations may be related to the variations in the characteristics of the study subjects, such as age and risk factors of cardiovascular disorders [34]. In the present study, a major portion of the study participants was hypertensive and diabetic and the high prevalence of microalbuminuria in our study supports the evidence that microalbuminuria prevalence is higher in populations with diabetes and hypertension [35–37].

This study finding on the association between microalbuminuria and MetS is in line with the findings of previous studies [13, 16, 34]. The prevalence of microalbuminuria was significantly higher in subjects with the MetS than those without MetS in Chinese (12% vs 2.9%) [34], Japanese (20.8% vs 12.2%) [13] and US adults (13.7% vs 4.8%) [16]. With regard to the relationship of microalbuminuria with the components of MetS, our study results are in line with the findings of previous works [13, 14, 16, 34]. For example, in Chinese adults, microalbuminuria was significantly associated with elevated BP and plasma glucose but not with other components of the MetS [14, 34]. In Japanese adults, a strong relationship was observed for microalbuminuria with elevated BP and hyperglycemia [13]. In the US adults, microalbuminuria was also significantly associated with elevated BP and hyperglycemia but not with the other components of MetS [16]. However, in other studies, microalbuminuria has been found to be associated with all components of MetS [12, 15], and such findings in these studies might be a reason that the components of MetS were not properly adjusted. Nevertheless, in individuals without existing cardiovascular disorders, such as treated hypertension could also be correlated with microalbuminuria [34].

In previous studies, an association was observed between hypertension and microalbuminuria [32, 38]. However, the mechanism for the association of microalbuminuria with hyperglycemia and elevated BP is not clear yet. In clinical studies, microalbuminuria has been demonstrated as an indicator of vascular damage and endothelial dysfunction [27]. Imbalanced metabolism of blood lipids and glucose are closely related to the development of MetS [2]. A recent study showed that MetS contributes to arterioles disease [39]. Peripheral circulation is thought to be the main determinant of BP and glucose regulation [34]. The MetS is proposed to be a disease of the periphery, which may be involved in the elevation of BP and hyperglycemia [34]. A study of the Korean population reported that individuals with microalbuminuria had higher blood glucose levels than individuals without microalbuminuria [40].

There are some limitations to this study. First, the cross-sectional nature of the data may preclude the causality between microalbuminuria and MetS being assumed. Second, the sample size was relatively small. Third, ACR was measured once in the collected morning urine. However, the high diagnostic precision of a spot urine collection could reduce the doubt related to inter-day variations in the rate of albumin excretion. Measurement of ACR in spot urine offers proper estimation in the epidemiologic surveys and has been used in many other previous studies [16, 30, 32, 41].

## Conclusions

This study reports the prevalence of microalbuminuria in Bangladeshi adults and this prevalence is more frequent in subjects with MetS, mainly attributable to high blood glucose and elevated BP. Moreover, the present study indicates a strong association between microalbuminuria and MetS in Bangladeshi adults. Further longitudinal studies are required to explore the relationship between microalbuminuria and MetS in diabetic and hypertensive individuals.

## Data Availability

The datasets used and analyzed during the present study are available from the corresponding author on reasonable request.

## Abbreviations

MetS: Metabolic syndrome
BMI: Body mass index
TG: Triglycerides
TC: Total cholesterol
LDL: Low-density lipoprotein
HDL: High-density lipoprotein
WHO: Word Health Organization
